# Progressive role of artificial intelligence in treatment decision-making in the field of medical oncology

**DOI:** 10.3389/fmed.2025.1533910

**Published:** 2025-02-13

**Authors:** Archana Reddy Bongurala, Dhaval Save, Ankit Virmani

**Affiliations:** ^1^Pediatrics – Omni Family Health, Bakersfield, CA, United States; ^2^Internal Medicine, Methodist Medical Center of Illinois, Peoria, IL, United States; ^3^Department of Artificial Intelligence, Virufy Inc., Los Altos, CA, United States

**Keywords:** artificial Intelligence, medical oncology, precision medicine, data analysis, personalized medicine, pediatric oncology

## Abstract

This article explores the role of artificial intelligence (AI) in medical oncology, emphasizing its impact on treatment decision-making for adult and pediatric cancer care. AI applications, including advanced imaging, drug discovery, and clinical decision support systems, enhance precision, personalization, and efficiency. Pediatric oncology benefits from improved diagnostics, risk stratification, and targeted therapies, despite unique challenges. AI-driven personalized medicine optimizes treatment strategies, improving patient outcomes and reducing costs. Ethical considerations, such as data privacy, algorithmic bias, and explainability, remain critical for responsible AI integration. Future advancements, including explainable AI and quantum computing, promise to redefine cancer care through data-driven insights.

## 1 Introduction

The integration of artificial intelligence (AI) into medical oncology marks a pivotal moment in cancer care. What began as basic rule-based systems has evolved into sophisticated deep learning platforms, fundamentally altering how oncologists approach treatment decisions (1). This transformation is not merely technological; it reflects a profound change in the utilization of data-driven insights for clinical decision-making.

AI and Machine Learning (ML) are significantly revamping the trajectory of medical oncology. They demonstrate the potential to advance treatment strategies by offering precision, personalization, and efficiency (2). By analyzing vast, complex datasets, AI algorithms can delve into the intricate details of tumor genomics, proteomics, and other molecular profiles, providing a deeper understanding of the underlying mechanisms driving cancer (2).

This article explores the expanding role of AI in both adult and pediatric oncology treatment, examining the current applications and future possibilities.

## 2 Evolution of AI systems in oncology

Early AI applications in oncology were limited to basic statistical analysis and straightforward decision making, capable of processing only a handful of variables. While these systems were groundbreaking for their time, they offered relatively simple support for treatment planning (1).

As computing power increased and machine learning algorithms advanced, the second generation of AI systems emerged in the early 2000s (3). These systems were capable of recognizing complex patterns and analyzing multiple factors simultaneously, significantly enhancing their ability to support clinical decision-making (3).

Today’s AI systems represent a quantum leap forward. Incorporating high-tech techniques such as deep learning, natural language processing, and real-time analytics, these systems provide comprehensive decision support across all aspects of cancer care, from diagnosis and treatment planning to monitoring and prognosis.

## 3 The landscape of AI in medical oncology treatment

### 3.1 Current applications in treatment selection for adult cancer

**FIGURE 1:**
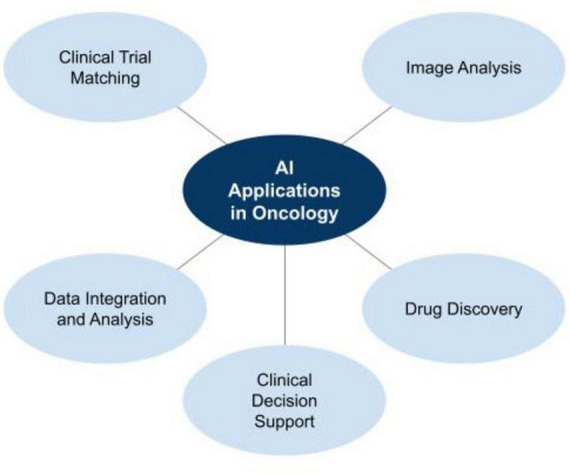
AI applications in oncology.

AI has emerged as an elevating force in adult oncology, advancing traditional approaches to cancer diagnosis, treatment, and monitoring. As shown in Figure 1, by integrating sophisticated algorithms and machine learning techniques, AI-powered tools are driving innovations across multiple therapeutic domains.

#### 3.1.1 Image analysis and treatment planning

One of the most prominent applications of AI in oncology is in the field of medical imaging. Computer-aided detection (CAD) systems, powered by modern machine learning algorithms, have increased the accuracy and efficiency of tumor detection in various imaging modalities, such as computed tomography (CT), magnetic resonance imaging (MRI), and radiography (4). Additionally, radiomics, a field that extracts quantitative features from medical images, has enabled the detailed analysis of tumor characteristics, including morphology, texture, and volumetric measurements. These features serve as useful predictors of tumor behavior and response to therapy. Furthermore, AI algorithms have upended radiation therapy planning by enhancing treatment delivery while minimizing damage to healthy tissues.

#### 3.1.2 Drug discovery and development

In the pharmaceutical domain, AI has accelerated drug discovery and development. Virtual screening methodologies use AI to expedite the evaluation of vast chemical libraries, reducing the time required to identify promising drug candidates (5). Predictive modeling systems, powered by machine learning algorithms, forecast drug efficacy and toxicity profiles, enabling researchers to prioritize compounds with the highest potential for therapeutic benefit and minimal adverse effects. Moreover, AI-driven personalized medicine has enabled the precise analysis of patient genetic profiles, facilitating the selection of optimal drug therapies and dosage regimens tailored to individual patients’ needs (5).

#### 3.1.3 Clinical decision support systems (CDSS)

Clinical decision support systems (CDSS) represent another critical application of AI in oncology. These systems provide real-time guidance to clinicians by synthesizing current clinical guidelines, patient-specific data, and emerging research findings. AI-powered risk stratification mechanisms enable the identification of patients at high risk for disease progression or adverse events, allowing for early intervention and proactive management (6). Additionally, CDSS contribute to treatment rationalization by incorporating multiple variables, including patient comorbidities, potential drug interactions, and therapeutic response patterns (6).

#### 3.1.4 Data integration and analysis

Modern AI systems have the capacity to integrate and analyze diverse data sources, including conventional clinical data and sophisticated molecular data (7). This comprehensive data integration enables real-time analysis, reforming the approach to treatment planning and patient monitoring in clinical oncology. By utilizing AI-powered insights, clinicians can make more informed decisions and tailor treatment plans to individual patient needs (7).

#### 3.1.5 Clinical trial matching

In the area of clinical research, AI has strengthened the efficiency of clinical trial matching processes. AI-powered systems can rapidly analyze vast quantities of medical data to identify optimal matches between patients and clinical trials, considering factors such as cancer subtype, genetic profile, and other relevant clinical characteristics (8). This upgraded matching process accelerates patient recruitment and increases the overall success rate of clinical trials in oncology.

### 3.2 Unique challenges and opportunities for pediatric oncology

The application of AI in pediatric oncology presents a unique and complex landscape. While it shows promising potential in cancer care, its implementation in the pediatric setting poses specific challenges. The relative rarity of pediatric malignancies, the dynamic nature of developmental genetics in children, and the stringent ethical guidelines governing pediatric research all contribute to a specialized context that differs significantly from adult oncology (9). Despite these challenges, AI technologies hold immense promise in pediatric cancer care.

#### 3.2.1 Improved diagnosis

In diagnostics, AI algorithms can analyze complex pediatric tumor characteristics, leading to earlier detection and more accurate diagnoses. This is particularly critical in pediatric oncology, where early intervention is often crucial for optimal outcomes (9).

#### 3.2.2 Risk stratification for hereditary cancers

AI also plays a vital role in risk stratification for hereditary cancers. By analyzing genetic data, it can identify children at increased risk of specific malignancies, enabling proactive surveillance and preventive interventions. This capability is particularly useful in managing families with known cancer syndromes and in identifying previously unrecognized hereditary patterns (9).

#### 3.2.3 Novel therapies and personalized treatment

In the therapeutic domain, AI is accelerating the development of novel treatment modalities specifically designed for pediatric cancers. It can analyze genetic and molecular data to identify unique therapeutic targets for pediatric cancers (9).

### 3.3 AI-powered precision oncology: a new era in cancer care

The powerful combination of AI and personalized medicine in the field of oncology offers unprecedented opportunities to tune-up patient care and improve treatment outcomes.

AI-driven individualized treatment planning is a significant development in personalized oncology. By analyzing a patient’s unique genetic and molecular profile, it can generate highly specific treatment recommendations. This precision medicine approach maximizes efficacy while minimizing adverse effects, surpassing traditional, one-size-fits-all treatment protocols (10).

The integration of AI-guided personalized medicine can improve patient outcomes. By customizing treatments to individual patient characteristics, this approach can lead to better survival rates and enhanced quality of life (10). Additionally, it can reduce the physical and psychological burden of cancer treatment.

From an economic perspective, AI-driven personalized medicine can reduce healthcare costs. By identifying optimal therapeutic strategies early on, this approach can minimize the financial burden associated with ineffective or poorly tolerated treatments. Moreover, it can optimize the utilization of healthcare resources (11).

#### 3.3.1 Emerging trends of computer vision in oncology

Computer vision is revolutionizing oncology by enabling AI-assisted procedures with significant potential (12). Real-time biopsy guidance systems, utilizing image analysis, can pinpoint suspicious tissue with greater accuracy, leading to more targeted biopsies and faster diagnoses. Furthermore, precision-guided surgical interventions, powered by computer vision algorithms, allow surgeons to visualize tumors and surrounding critical structures with unprecedented clarity, minimizing invasiveness and improving surgical outcomes (13). Automated tissue analysis, utilizing deep learning techniques, can rapidly analyze microscopic images, identifying subtle patterns and features that may be missed by human pathologists, leading to more accurate and efficient cancer diagnoses and prognosis predictions (14). These advancements hold the promise of transforming cancer care by improving diagnostic accuracy, enhancing surgical precision, and ultimately improving patient outcomes.

### 3.4 Ethical considerations and challenges

While AI offers immense potential, it is essential to address ethical considerations and challenges to ensure its responsible and beneficial use in oncology:

• **Data Privacy and Security:** Protecting patient privacy and data security is paramount when analyzing sensitive health information (15).
• **Algorithmic Bias:** AI algorithms must be trained on diverse and representative datasets to avoid biases that could lead to unfair treatment decisions.
• **Explainability:** AI models need to be interpretable to build trust among healthcare providers and patients.
• **Regulatory Framework:** Clear regulatory guidelines are needed to ensure the safe and effective use of AI in healthcare (15).

### 3.5 The future of AI in medical oncology decision-making

The integration of AI into oncology decision-making is poised to enrich cancer care. One of the most significant ascents in this field is the integration of AI with Electronic Health Records (EHRs) (16). This integration will enable real-time analysis of patient data, facilitating the development and implementation of dynamic treatment strategies. By continuously monitoring patient parameters and response patterns, clinicians can make timely adjustments to treatment plans (16).

To ensure trust and transparency in AI-driven decision-making, the development of Explainable AI (XAI) is crucial. XAI technologies provide clinicians with clear insights into the reasoning behind AI-generated recommendations, empowering them to make informed decisions and maintain oversight of patient care (17).

Emerging technologies like quantum computing and advanced visualization tools are known to significantly expand the capabilities of AI systems (18). The development of autonomous systems with self-learning capabilities will likely lead to even more sophisticated decision support tools. Moreover, the integration of AI with virtual tumor boards and remote consultation platforms will improve the access to expert-level care, even in remote locations (18). As technology continues to evolve, we can anticipate even more advanced AI systems capable of processing increasingly complex datasets and delivering highly precise recommendations.

**FIGURE 2:**
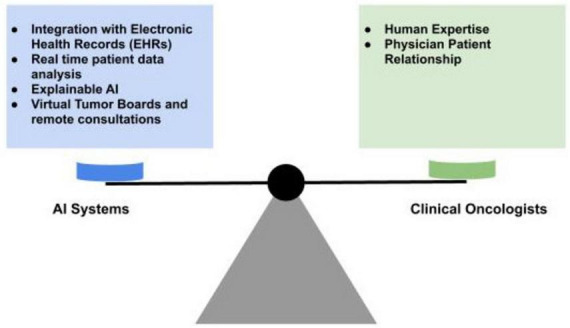
Future of AI in medical oncology: balancing AI and human expertise in oncology.

As shown in Figure 2, the future of oncology care envisions a collaborative partnership between AI systems and clinical oncologists (16). AI will serve as a powerful decision support tool, augmenting human expertise with data-driven insights. By combining the computational power of AI with the clinical expertise of oncologists, this collaborative approach will optimize patient care and improve outcomes (16).

This synergistic relationship between AI and human expertise will redefine the field of oncology. AI will fine-tune the quality and efficiency of cancer care while preserving the essential human elements of medical practice. This balanced approach ensures that technological advancements support and boost, rather than replace, the fundamental physician-patient relationship. The ultimate goal remains steadfast; to provide optimal care for cancer patients through the intelligent application of both artificial and human intelligence.

## 4 Conclusion

The progressive integration of AI into oncology decision-making represents a paradigm shift in cancer care. As these systems continue to evolve, they will likely play an increasingly important role in improving patient outcomes, standardizing care quality, and enhancing our understanding of cancer treatment. The key to success lies in thoughtful implementation that maintains the essential balance between technological capability and human expertise.

## Data Availability

The original contributions presented in this study are included in this article/supplementary material, further inquiries can be directed to the corresponding author.

## References

[1] Vobugari N Raja V Sethi U Gandhi K Raja K Surani S. Advancements in oncology with artificial intelligence-A review article. *Cancers (Basel).* (2022) 14:1349. 10.3390/cancers14051349 PMC8909088 35267657

[2] Rajdeo P Aronow B Surya Prasath V. Deep learning-based multimodal spatial transcriptomics analysis for cancer. *Adv Cancer Res.* (2024) 163:1–38. 10.1016/bs.acr.2024.08.001 39271260 PMC11431148

[3] Kaul V Enslin S Gross S. History of artificial intelligence in medicine. *Gastrointest Endosc.* (2020) 92:807–12. 10.1016/j.gie.2020.06.040 32565184

[4] Gao S Xu Z Kang W Lv X Chu N Xu S Artificial intelligence-driven computer aided diagnosis system provides similar diagnosis value compared with doctors’ evaluation in lung cancer screening. *BMC Med Imaging.* (2024) 24:141. 10.1186/s12880-024-01288-3 PMC11165751 38862884

[5] Paul D Sanap G Shenoy S Kalyane D Kalia K Tekade R. Artificial intelligence in drug discovery and development. *Drug Discov Today.* (2021) 26:80–93. 10.1016/j.drudis.2020.10.010 33099022 PMC7577280

[6] Amann J Vetter D Blomberg S Christensen H Coffee M Gerke S Z-Inspection initiative. To explain or not to explain?-Artificial intelligence explainability in clinical decision support systems. *PLoS Digit Health.* (2022) 1:e0000016. 10.1371/journal.pdig.0000016 PMC9931364 36812545

[7] Lipkova J Chen R Chen B Lu M Barbieri M Shao D Artificial intelligence for multimodal data integration in oncology. *Cancer Cell.* (2022) 40:1095–110. 10.1016/j.ccell.2022.09.012 36220072 PMC10655164

[8] Chow R Midroni J Kaur J Boldt G Liu G Eng L Use of artificial intelligence for cancer clinical trial enrollment: A systematic review and meta-analysis. *J Natl Cancer Inst.* (2023) 115:365–74. 10.1093/jnci/djad013 36688707 PMC10086633

[9] Yang Y Zhang Y Li Y. Artificial intelligence applications in pediatric oncology diagnosis. *Explor Target Antitumor Ther.* (2023) 4:157–69. 10.37349/etat.2023.00127 36937318 PMC10017189

[10] Bhinder B Gilvary C Madhukar N Elemento O. Artificial intelligence in cancer research and precision medicine. *Cancer Discov.* (2021) 11:900–15. 10.1158/2159-8290.CD-21-0090 33811123 PMC8034385

[11] Chen Z Lin L Wu C Li C Xu R Sun Y. Artificial intelligence for assisting cancer diagnosis and treatment in the era of precision medicine. *Cancer Commun (Lond).* (2021) 41:1100–15. 10.1002/cac2.12215 34613667 PMC8626610

[12] Olveres J González G Torres F Moreno-Tagle J Carbajal-Degante E Valencia-Rodríguez A What is new in computer vision and artificial intelligence in medical image analysis applications. *Quant Imaging Med Surg.* (2021) 11:3830–53. 10.21037/qims-20-1151 34341753 PMC8245941

[13] Ardila C González-Arroyave D. Precision at scale: Machine learning revolutionizing laparoscopic surgery. *World J Clin Oncol.* (2024) 15:1256–63. 10.5306/wjco.v15.i10.1256 39473862 PMC11514504

[14] Zhu M Sali R Baba F Khasawneh H Ryndin M Leveillee R Artificial intelligence in pathologic diagnosis, prognosis and prediction of prostate cancer. *Am J Clin Exp Urol.* (2024) 12:200–15. 10.62347/JSAE9732 39308594 PMC11411179

[15] MacIntyre M Cockerill R Mirza O Appel J. Ethical considerations for the use of artificial intelligence in medical decision-making capacity assessments. *Psychiatry Res.* (2023) 328:115466. 10.1016/j.psychres.2023.115466 37717548

[16] Cabral B Braga L Syed-Abdul S Mota F. Future of artificial intelligence applications in cancer care: A global cross-sectional survey of researchers. *Curr Oncol.* (2023) 30:3432–46. 10.3390/curroncol30030260 36975473 PMC10047823

[17] Laios A De Jong D Kalampokis E. Beauty is in the explainable artificial intelligence (XAI) of the “agnostic” beholder. *Transl Cancer Res.* (2023) 12:226–9. 10.21037/tcr-22-2664 36915578 PMC10007889

[18] Solenov D Brieler J Scherrer J. The potential of quantum computing and machine learning to advance clinical research and change the practice of medicine. *Mo Med.* (2018) 115:463–7. 30385997 PMC6205278

